# UBE2N promotes cell viability and glycolysis by promoting Axin1 ubiquitination in prostate cancer cells

**DOI:** 10.1186/s13062-024-00469-y

**Published:** 2024-05-07

**Authors:** Bo Yang, Weihua Chen, Tianyi Tao, Jun Zhang, Dehui Kong, Jidong Hao, Chao Yu, Guoqiang Liao, Hua Gong

**Affiliations:** 1 Department of Urology, Shanghai University of Medicine & Health Sciences Affiliated Zhoupu Hospital, No.1500 Zhouyuan Road, Pudong New Area, Shanghai, 201318 China; 2 Department of Urology, Shanghai East Hospital, Tongji University, Shanghai, 200120 China; 3 Graduate School, Shanghai University of Traditional Chinese Medicine, Shanghai, 201203 China; 4 Experimental Cellular Therapy Group, University of California, San Francisco, San Francisco, 94103 USA; 5 Department of Urology, Longhua Hospital, Shanghai University of Traditional Chinese Medicine, Shanghai, 200032 China

**Keywords:** Axin1, Glycolysis, Prostate cancer, UBE2N, Ubiquitination, Wnt/β-catenin

## Abstract

**Background:**

Ubiquitin-conjugating enzyme E2 N (UBE2N) is recognized in the progression of some cancers; however, little research has been conducted to describe its role in prostate cancer. The purpose of this paper is to explore the function and mechanism of UBE2N in prostate cancer cells.

**Methods:**

UBE2N expression was detected in Cancer Genome Atlas Prostate Adenocarcinoma (TCGA-PRAD) data, prostate cancer tissue microarrays, and prostate cancer cell lines, respectively. UBE2N knockdown or overexpression was used to analyze its role in cell viability and glycolysis of prostate cancer cells and tumor growth. XAV939 or Axin1 overexpression was co-treated with UBE2N overexpression to detect the involvement of the Wnt/β-catenin signaling and Axin1 in the UBE2N function. UBE2N interacting with Axin1 was analyzed by co-immunoprecipitation assay.

**Results:**

UBE2N was upregulated in prostate cancer and the UBE2N-high expression correlated with the poor prognosis of prostate cancer. UBE2N knockdown inhibited cell viability and glycolysis in prostate cancer cells and restricted tumor formation in tumor-bearing mice. Wnt/β-catenin inhibition and Axin1 overexpression reversed the promoting viability and glycolysis function of UBE2N. UBE2N promoted Axin1 ubiquitination and decreased Axin1 protein level.

**Supplementary Information:**

The online version contains supplementary material available at 10.1186/s13062-024-00469-y.

## Significance

Abnormally high expression of UBE2N acts as a cancer promoter to strengthen glycolysis and viability of prostate cancer cells. The mechanism of UBE2N is its promotion of the ubiquitination of Axin1.

**Supplementary Information:**

The online version contains supplementary material available at 10.1186/s13062-024-00469-y.

## Introduction

Prostate cancer is a common malignancy for universal men and causes millions of cancer-related deaths although treatment strategies have improved [1]. Recent researches on cancer management suggest that understanding the biological survival of cancer cells provides an opportunity for a more effective diagnosis, treatment, and prognosis. Glycolysis, a process that provides the energy and substances for cancer cell proliferation, is recognized as a therapy targeting various cancers and also can be used for the prevention of prostate cancer progression [2]. Therefore, the identification of novel glycolysis-associated molecular markers provides a perspective target for prostate cancer treatment.

Ubiquitination is a common post-transcriptional modification and is essential for the regulation of the degradation of target proteins and the maintenance of cell homeostasis. Its dysregulation is involved in the pathological process of diseases [3]. Increasing works have proved that ubiquitination or deubiquitination modification on oncogenes or tumor suppressor genes plays a crucial role in tumor progression [4]. UBE2N (also called Ubc13) is a membrane of E2 ubiquitin-conjugating enzymes and is responsible for the synthesis of Lysine 63-linked polyubiquitination chains [5]. Recent studies have characterized UBE2N as a crucial growth promoter of some human tumors, such as ovarian cancer and acute myeloid leukemia [6, 7], which indicates that UBE2N is an oncogene and potential therapeutic target for cancers. However, little is known about the physiological function of UBE2N in prostate cancer, and the possible mechanism of UBE2N in the process of tumor progression needs further investigation.

Wnt/β-catenin signaling pathway is conserved across species in regulating many biological processes (e.g., cell fate determination, cell differentiation, and cell proliferation) [8]. Deregulation of the Wnt/β-catenin pathway occurs in various kinds of cancer as well as in prostate cancer as suggested by genome sequencing and gene expression analyses [6, 9]. As reported in previous studies, activation of Wnt/β-catenin signaling or its target genes such as c-myc in prostate cancer promoted tumor progression, while inactivating Wnt/β-catenin signaling restricted tumor growth [10, 11]. The degradation of β-catenin by a dedicated cytoplasmic destruction complex is the key regulatory step of Wnt/β-catenin signaling. In this destruction complex, Axin1 serves as a central scaffold protein and cooperates with three other core components, the adenomatous polyposis coli (APC), casein kinase-1 (CK1), and kinases glycogen synthase kinase-3 alpha/beta (GSK-3) [12, 13]. In cancer, proteasomal hydrolysis of Axin1 represented its weakened Wnt-regulated function and cytosolic β-catenin accumulation [12, 14]. Targeting Axin1 deubiquitination or inducing Axin1 expression portended an anticancer strategy in some cancers [13, 15]. Although genetic alterations of APC, Axin1 and β-catenin have been detected in clinical specimens of advanced prostate cancer [16], the interaction between Axin1 and Wnt/β-catenin pathway in affecting prostate cancer progression is poorly understood.

Some E3 ubiquitin ligases increase the ubiquitination of Axin1 and therefore facilitate β-catenin entry into the nucleus had been proven in promoting the progression of colorectal cancer and gastric cancer [14, 17, 18]. However, few works have been conducted to clarify the role of UBE2N in Axin1 stability and Wnt/β-catenin pathway activation, especially in prostate cancer. In this study, we first analyzed RNA sequencing data for the mRNA expression of UBE2N from the TCGA database and the relative mRNA and protein expression of UBE2N in prostate cancer patients’ tissues and tumor cell lines. Results showed that UBE2N was much higher in prostate cancer and tumor cells than in adjacent normal tissues and normal prostatic epithelial cell lines. Therefore, we further investigated the efect of UBE2N on the development of prostate cancer. Our research indicated that UBE2N promoted cell viability and glycolysis dependent on the β-catenin pathway activation via ubiquitination and degradation of Axin1. The oncogenic properties of UBE2N were proved in xenograft mice to promote tumor growth and cell proliferation, making it a potential target for treatment for prostate cancer and dramatically improve patient survival.

## Materials and methods

### Bioinformatics analysis

UBE2N mRNA level was examined in the gene expression data of the prostate adenocarcinoma (PRAD) cohort in The Cancer Genome Atlas (TCGA). GSEA version 2.0 was run to analyze gene set enrichment analysis (GSEA) of pathways and genes and excluded gene sets less than 10. The most significant pathways associated with UBE2N expression were examined by the permutation test 1000 times, and the cut-off value of the *P*-value was set at 0.01.

### Clinical specimens

Ninety tissue microarrays of prostate cancer and 30 normal prostate tissue microarrays were purchased from Shanghai Outdo Biotech. Patients who had received any treatment or biological medication before sampling were excluded from the study.

### Immunohistochemistry (IHC)

The anti-UBE2N antibody (ab109286; Abcam) and anti-Axin1 antibody (ab133221; Abcam) were applied to the paraffin-embedded sections followed by the secondary antibody (D-3004, Shanghai Long Island Biotec. Co. Ltd). Immunoreactivity was scored using the H-score system by two investigators based on staining intensity (0, negative; 1, weak; 2, moderate; 3, strong) and percentage of positive cells (0, < 5%; 1, 5–25%; 2, 25–50%; 3, 50–75%; 4, > 75%). With an IHC score = 6 as the cutoff point, patients were categorized into low- and high-expression groups.

### Cell culture

The human prostatic epithelial cell line (HPEpic) was obtained from XinYu Bio-Technology, Shanghai, China. Four prostate cancer cell lines 22RV1, PC3, LNCaP and DU145, and a 293T cell line were provided by the Shanghai Cell Bank (Shanghai, China). RPMI-1640 and DMEM with a mixture of 10% fetal bovine serum (Gibco, Grand Island, NY, USA), penicillin (100 units/ml) and streptomycin (100 µg/ml) were used for cell culture and the cultural condition was 37 °C and 5% CO_2_/95% air atmosphere. To investigate the role of UBE2N in process of Axin1 protein synthesis, PC3 cells were treated with 10 µM proteasome inhibitor MG132 (S2619; Selleck). To investigate the role of Wnt/β-catenin in UBE2N-mediated prostate cancer progression, PC3 cells were treated with 10 µM XAV939 (S1180; Selleck).

### Lentivirus-mediated RNA knockdown and overexpression of UBE2N

UBE2N knockdown in cells was achieved by lentivirus-mediated RNA interference sequencing transfection. The RNA interference sequence for UBE2N was synthesized and cloned into pLKO.1 vector. The specific three interference sequences were listed: shUBE2N-1, 5′-GGAAGAATATGTTTAGATA-3′; shUBE2N-2, 5′-GCAGTGGAAGACCAACGAA-3′; and shUBE2N-3, 5′-GCACAGTTCTGCTATCGAT-3′. Scramble shRNA (5′-GGAATGATGATAGATATTA-3′) was used as negative control (shNC).

UBE2N overexpression vector was constructed by cloning the coding sequence of UBE2N into pLVX-puro expression vector. In presence of lipofectamine 2000 (Invitrogen, USA), the constructs and the packaging plasmids psPAX2 and pMD2G were co-transfected into 293T cells. At 48 h after transfection, the cell culture medium containing the viral particles was collected to infect prostate cancer cell lines. Blank pLVX-puro vector was used as negative control.

### Axin1 overexpression

The full-length human Axin1 was amplified and cloned into the pcDNA3.1 vector. To overexpress Axin1, the pcDNA3.1 vector expressing Axin1 was transfected into prostate cancer cell lines using Lipofectamine 2000 (Invitrogen, USA). Blank pcDNA3.1 vector was used as negative control.

### Cell viability evaluation by CCK-8 assay

Cells were plated in a 96-well plate (5 × 10^3^ cells/well) and cultured overnight at 37˚C. After 48 h treatment mentioned above, each well received 10 µL of CCK-8 solution (Invitrogen). By measuring absorbance at 450 nm with a microplate reader (Invitrogen), absorbance was determined after an hour of incubation.

### Extracellular flux analysis

A Seahorse XF24 Extracellular Flux Analyzer determined cellular oxygen consumption rates (OCR) and extracellular acidification rates (ECAR) as previously described [19]. Briely, cells digested to a density of 1 × 10^4^/well, were seeded in XF24 culture plates (Agilent Technologies, Santa Clara, CA, USA), and were then placed in an incubator of 37 °C and 5% CO_2_ for 24 h. Around 1 h before detection, cells were shifted into an incubator without CO_2_, and culture medium was replaced by XF Base Medium (Agilent Technologies). OCR was measured using Seahorse XF Cell Mito Stress Test Kit (103015-100; Agilent Technologies) and ECAR was measured using Seahorse XF Glycolytic Rate Assay Kit (103344-100; Agilent Technologies).

### Measurement of lactate and adenosine triphosphate (ATP) production

Lactate production was measured using a Lactic Acid assay kit (A019-2; Nanjing Jiancheng Bioengineering Institute, China) following protocols of the manufacturer’s instruction. ATP content was measured with the ATP assay kit (A095; Nanjing Jiancheng Bioengineering Institute), as per the manufacturer’s protocol. ATP concentration was normalized to the corresponding total protein amounts from each sample.

### Quantitative RT-PCR (RT-qPCR)

Total RNA was isolated using Trizol reagent (Invitrogen, USA). The first-strand cDNA was synthesized using the PrimeScript RT Reagent Kit (RR047A; Takara Biomedical Technology (Beijing) Co., Ltd, China) as per manufacturer’s instructions. RT-qPCR was performed using SYBR Green PCR Master Mix (4,309,155; Thermo Fisher Scientific). The relative expression level of target genes was normalized to that of β-actin using the 2^−ΔΔCt^ method. Primers for PCR were as follows: UBE2N, forward 5′-AGTTCCTGGCATCAAAGC-3′ and reverse 5′- GGGGACCACTTATCTTTC-3′; Axin1, forward 5′-CAGTCAACCCCTATTATGTC-3′ and reverse 5′-GAACTTCTGAGGCTCCAC-3′; β-actin, forward 5′-AGGATTCCTATGTGGGCGAC-3′ and reverse 5′-ATAGCACAGCCTGGATAGCAA-3′.

### Western blot analysis

The total protein sample of whole cell lysates was isolated using a radioimmunoprecipitation buffer mixed with a proteinase inhibitor (Beyotime, China). The total protein sample of nuclear extracts was obtained using NE-PER™ Nuclear and Cytoplasmic Extraction Reagents (Thermo Fisher Scientific, USA). After quantification, equal quality of total protein samples was subjected to SDS-PAGE. The separated proteins were electroblotted from gel to nitrocellulose membranes (Millipore, USA). The membrane was blocked with 5% skim milk for 1 h. Target proteins in the membranes were immunoreacted with primary antibodies overnight at 4 °C. The membrane was then incubated with HRP-conjugated rabbit secondary antibody (Beyotime, Shanghai, China) at room temperature for 1 h. Signals of target genes were developed by an enhanced chemiluminescence system. Primary antibodies used in this experiment were as follows: Anti-UBE2N (ab25885; Abcam), anti-Axin1 (#3323; Cell Signaling Technology), anti-c-myc (#5605; Cell Signaling Technology), anti-HK2 (ab104836; Abcam), anti-PKM2 (ab85555; Abcam), anti-Histone H3 (ab1791; Abcam), anti-Ub (ab137031; Abcam), and anti-β-catenin (#8480; Cell Signaling Technology).

### Co-immunoprecipitation (Co-IP)

Whole lysates from samples were extracted using IP buffer. The lysates were then pre-cleaned with 25 µL of 50% protein A/G-agarose beads. The supernatant was incubated with anti-UBE2N antibody (#6999; Cell Signaling Technology) and anti-Axin1 antibody (#2087; Cell Signaling Technology) and protein A/G beads at 4 °C overnight to pull down target proteins. The tubes were centrifuged at 1,500 rpm for 60 s at 4 °C to collect immune complexes, which were then washed with 1 ml of IP lysis buffer and repeated five times. Bead-binding proteins were diluted using protein loading buffer, subjected to electrophoresis on SDS-PAGE gel, and blotted with the antibodies against anti-UBE2N antibody (ab25885; Abcam) and anti-Axin1 antibody (#3323; Cell Signaling Technology).

### Half-life of c-Myc

PC3 cells were infected with lentivirus expressing UBE2N for 24 h, then exposed to 0.1 mg/ml cycloheximide (CHX, Sigma-Aldrich). Cells were collected at 0, 3 or 6 h after exposure and subjected to immunoblotting.

### Tumor growth in a tumor-bearing mouse model

Experiments were performed according to the principles of the Committee on Ethics of Animal Experiments of Shanghai University of Medicine & Health Sciences Affiliated Zhoupu Hospital. A total of 2 × 10^6^ Hela cells transduced with pLKO.1-shUBE2N or pLKO.1-shNC were subcutaneously injected into male nude mice (4–6 weeks) (*n* = 6 each group). Tumor length and width monitoring was performed every three days from day 12 after cancer cell injection. Length and width values were used for tumor volume calculation based on an equation: 1/2 × length × width^2^ as previously described [20].

### Immunofluorescence microscopy

After fixing and permeabilizing, the tissues collected from the xenograft tumors were blocked with 1% bovine serum albumin in PBS for 30 min and incubated with anti-PCNA (10205-2-AP; Proteintech) and Alexa Fluor 488-labeled Goat Anti-Mouse IgG (H + L) (A0423; Beyotime Biotechnology) antibodies. DAPI staining was used to visualize cell nuclei. Visualization of positively stained cells was performed using a Leica DM2000 microscopy system (Leica Microsystems, Wetzlar, Germany).

### Statistical analysis

Data analysis and statistic was conducted in GraphPad Prism Software. Results are expressed as mean ± SD. Between-group differences were evaluated using Student’s *t*-test or ANOVA. The Kaplan–Meier method was used to analyze overall survival. *P* values of < 0.05 were considered to indicate statistical significance.

## Results

### Upregulation of UBE2N correlates with the poor prognosis of prostate cancer

**Fig. 1:**
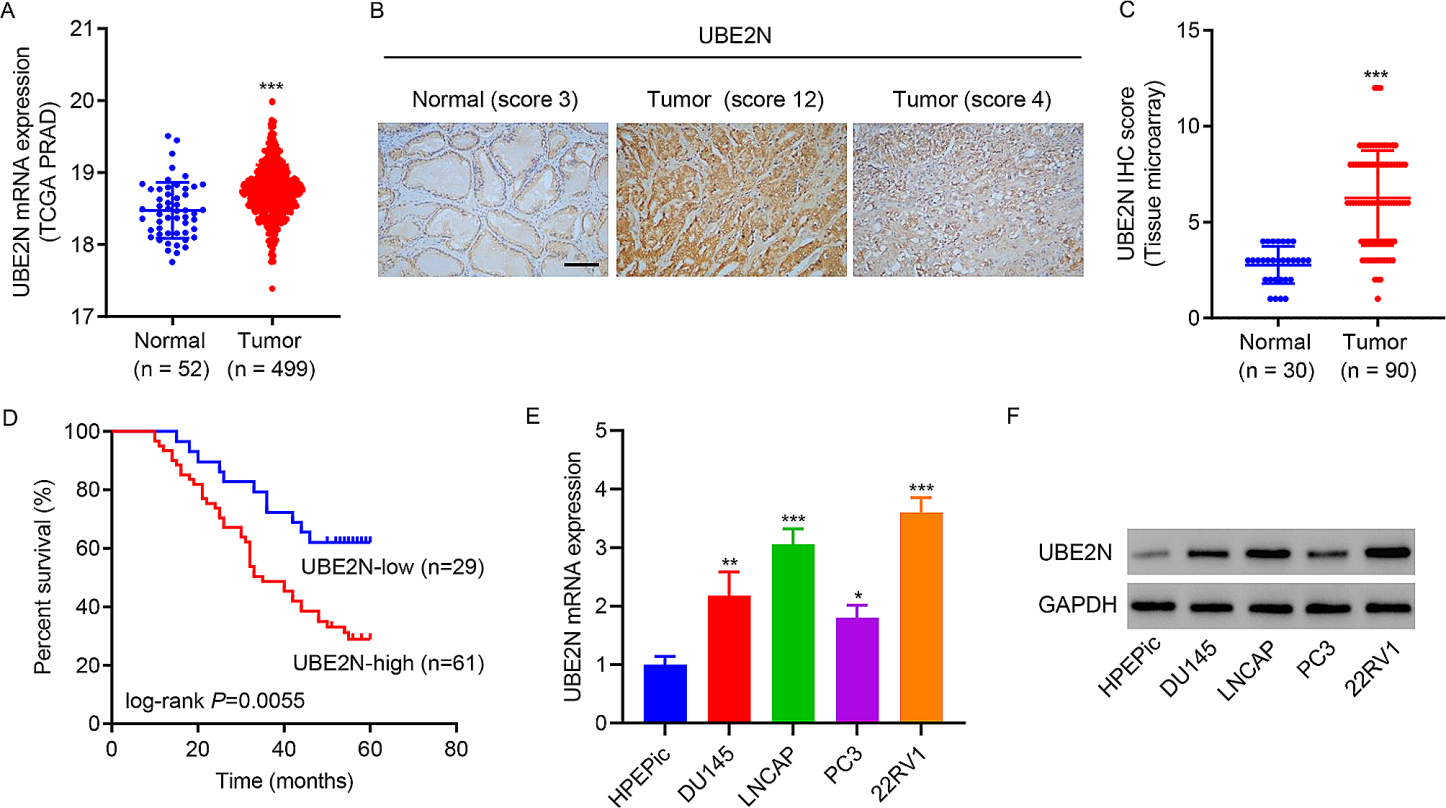
Upregulation of UBE2N correlates with the poor prognosis of prostate cancer. **(A)** UBE2N mRNA is expressed highly in tumor tissues in TCGA-PARD dataset. **(B, C)** Analysis of UBE2N protein in prostate cancer tissue microarray by IHC staining (scale bar, 100 μm). **(D)** Survival rate was compared between patients with high and low UBE2N levels by the Log-rank test. **(E)** UBE2N mRNA levels were examined using RT-qPCR (*n* = 3). **(F)** Western blot determined UBE2N protein expression (*n* = 3). Data are expressed as mean ± SD. *p < 0.05, **p < 0.01, ***p < 0.001 vs. normal or HPEPic

As the analysis in TCGA-PRAD data, UBE2N mRNA expression was elevated remarkably in tumor specimens when compared to normal tissues (Fig. 1A). UBE2N protein expression in prostate cancer tissue microarrays was examined by IHC staining (Fig. 1B). UBE2N protein expression was elevated remarkably in tumor specimens when compared to normal tissues (Fig. 1C). Correlation analysis between UBE2N and clinical characteristics displayed that UBE2N level correlated significantly with prostate specific antigen (PSA), T stage, and Gleason score (Table 1). Based on the UBE2N IHC score, 90 patients were grouped into a UBE2N-high expression subgroup (*n* = 61) and a UBE2N-low expression (*n* = 29) subgroup. The UBE2N-high expression subgroup correlated with a low survival rate as compared with the UBE2N-low expression subgroup (Fig. 1D). The result of the GSEA displayed the positive correlations between UBE2N expression and “HALLMARK_GLYCOLYSIS”, “KEGG_OXIDATIVE_PHOSPHORYLATION”, “REACTOME_SIGNALING_BY_WNT”, or “HALLMARK_MYC_TARGETS_V1” in patients with prostate cancer (Figure S1). The increases of UBE2N mRNA and protein expressions were also found in DU145, LNCAP, PC3, and 22RV1 prostate cancer cell lines (Fig. 1E and F).

**Table 1:** Correlation of tissue high and low levels of UBE2N with clinicopathological features of prostate cancer patients

Clinicopathological features	UBE2N		*P* value
	Low (*n* = 29)	High (*n* = 61)	
**Age (years)**			0.418
< 60 (*n* = 49)	14	35	
≥ 60 (*n* = 41)	15	26	
**PSA (ng/ml)**			0.019
< 4 (*n* = 34)	16	18	
≥ 4 (*n* = 56)	13	43	
**T stage**			0.020
T2 (*n* = 37)	17	20	
T3-4 (*n* = 53)	12	41	
**N stage**			0.634
N0 (*n* = 65)	20	45	
N1 (*n* = 25)	9	16	
**M stage**			0.380
M0 (*n* = 73)	22	51	
M1 (*n* = 17)	7	10	
**Gleason score**			0.029
3–6 (*n* = 35)	16	19	
7–10 (*n* = 55)	13	42	

PSA, prostate specific antigen. Differences between groups were done by the Chi-square test

### Knockdown of UBE2N inhibits cell viability and glycolysis in prostate cancer cells

**Fig. 2:**
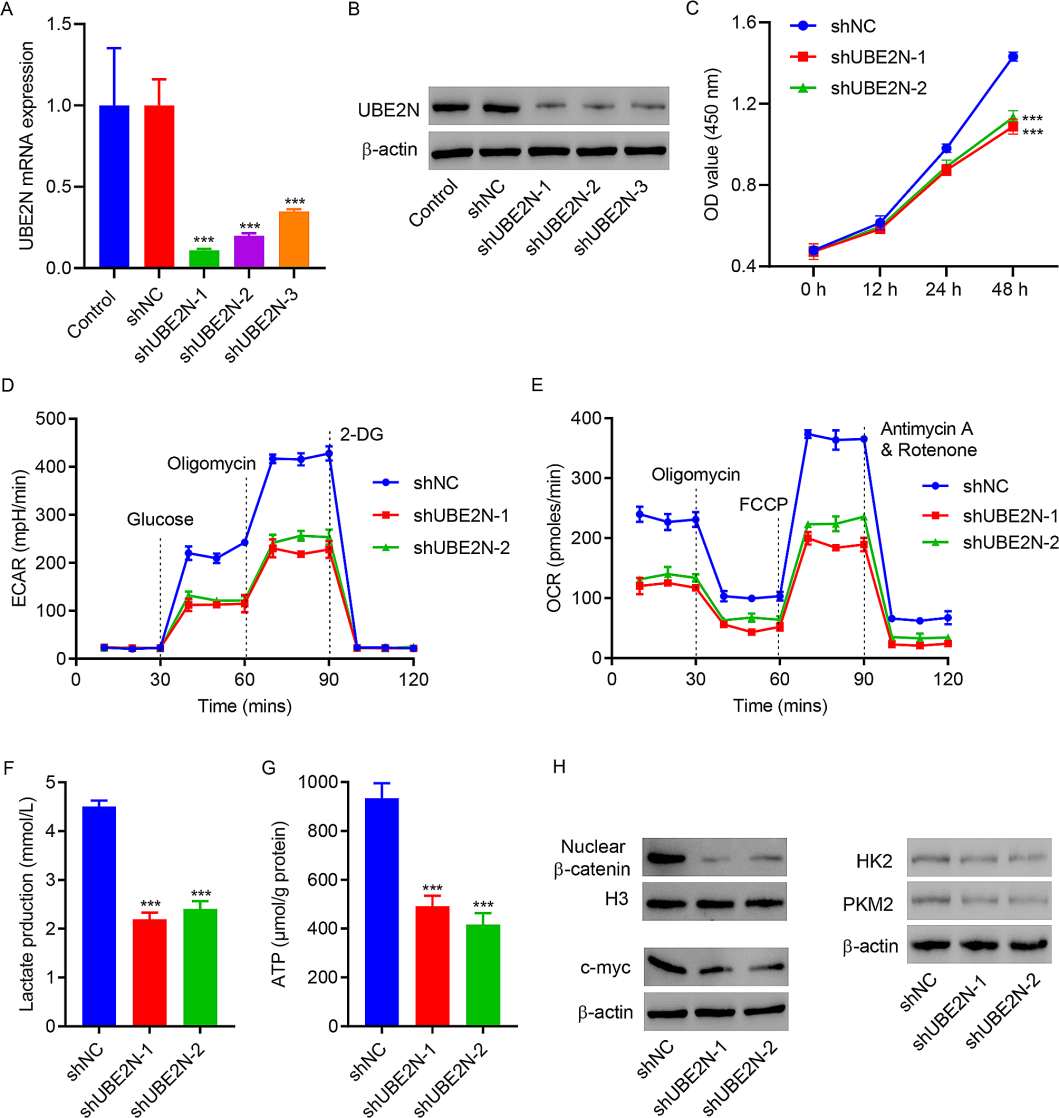
Knocking down of UBE2N inhibits viability and glycolysis of 22RV1 cells. **(A)** RT-qPCR and **(B)** Western blot assay showed UBE2N expression levels in 22RV1 cells transduced with either scramble shRNA (shNC) or shUBE2N-1, -2, and − 3. **(C) **CCK-8, **(D)** Extracellular acidification rate (ECAR), **(E)** oxygen consumption rate (OCR), **(F) **lactate, **(G)** ATP production, and **(H) **expression of c-myc, nuclear β-catenin, HK2 and PKM2. Data are expressed as mean ± SD (*n* = 3). ***p < 0.001 vs. shNC

To ascertain the function of UBE2N, UBE2N was knocked down in 22RV1 prostate cancer cells by cell transduction with UBE2N shRNA lentiviral vector (shUBE2N-1, -2, and − 3) (Fig. 2A and B). shUBE2N-1 and − 2 transduced 22RV1 cells were used for further analysis of cell viability and glycolysis. Figure 2C showed the decreased cell viability at 48 h in shUBE2N-1 and shUBE2N-2 transduced 22RV1 cells. Figure 2D and E showed decreased ECAR and OCR and Fig. 2F and G showed decreased lactate and ATP production in shUBE2N-1 and shUBE2N-2 transduced 22RV1 cells, suggesting knockdown of UBE2N suppresses glycolysis. Western blotting showed that knockdown of UBE2N reduced expression of c-myc, nuclear β-catenin and two glycolytic enzymes HK2 and PKM2 (Fig. 2H).

### Knockdown of UBE2N suppresses tumor formation in nude mice

**Fig. 3:**
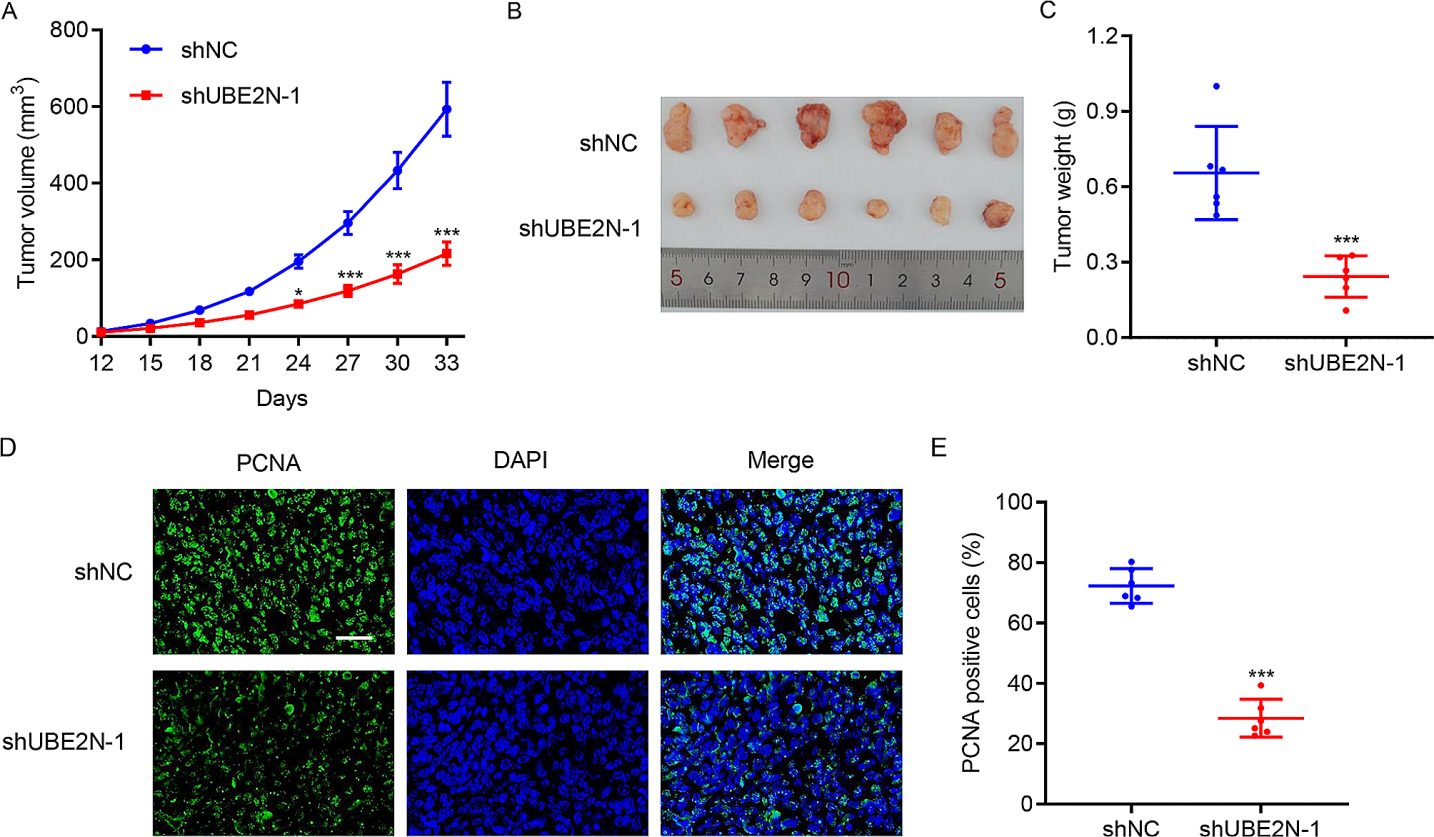
UBE2N knockdown suppresses xenograft growth in a tumor-bearing mouse model. The mouse was injected subcutaneously with 22RV1 cells transduced with either shNC or shUBE2N-1. **(A)** Tumor volume. On Day 33, xenografts were removed for **(B)** photographed and** (C)** weighted. **(D, E)** PCNA immunofluorescence staining (scale bar, 50 μm). Data are expressed as mean ± SD (*n* = 6). *p < 0.05, ***p < 0.001 vs. shNC

The role of the knockdown of UBE2N in tumor growth was determined in a mouse-bearing xenograft model with injection of 22RV1 cells expressing shUBE2N-1 or shNC. During tumor growth, the tumor volume of shUBE2N-xenograft was significantly less than shNC-xenograft (Fig. 3A). Xenografts were collected after 33 days and used to measure tumor size and weight. As shown in Fig. 3B and C, we observed the shUBE2N-xenograft tumor size and weight were smaller than the shNC-xenograft. Immunofluorescence showed that shUBE2N-xenograft presented decreased PCNA-positive cells than shNC-xenograft (Fig. 3D and E), suggesting that knockdown of UBE2N suppresses prostate cancer tumor growth in vivo.

#### UBE2N functions through the Wnt/β-catenin signaling pathway

**Fig. 4:**
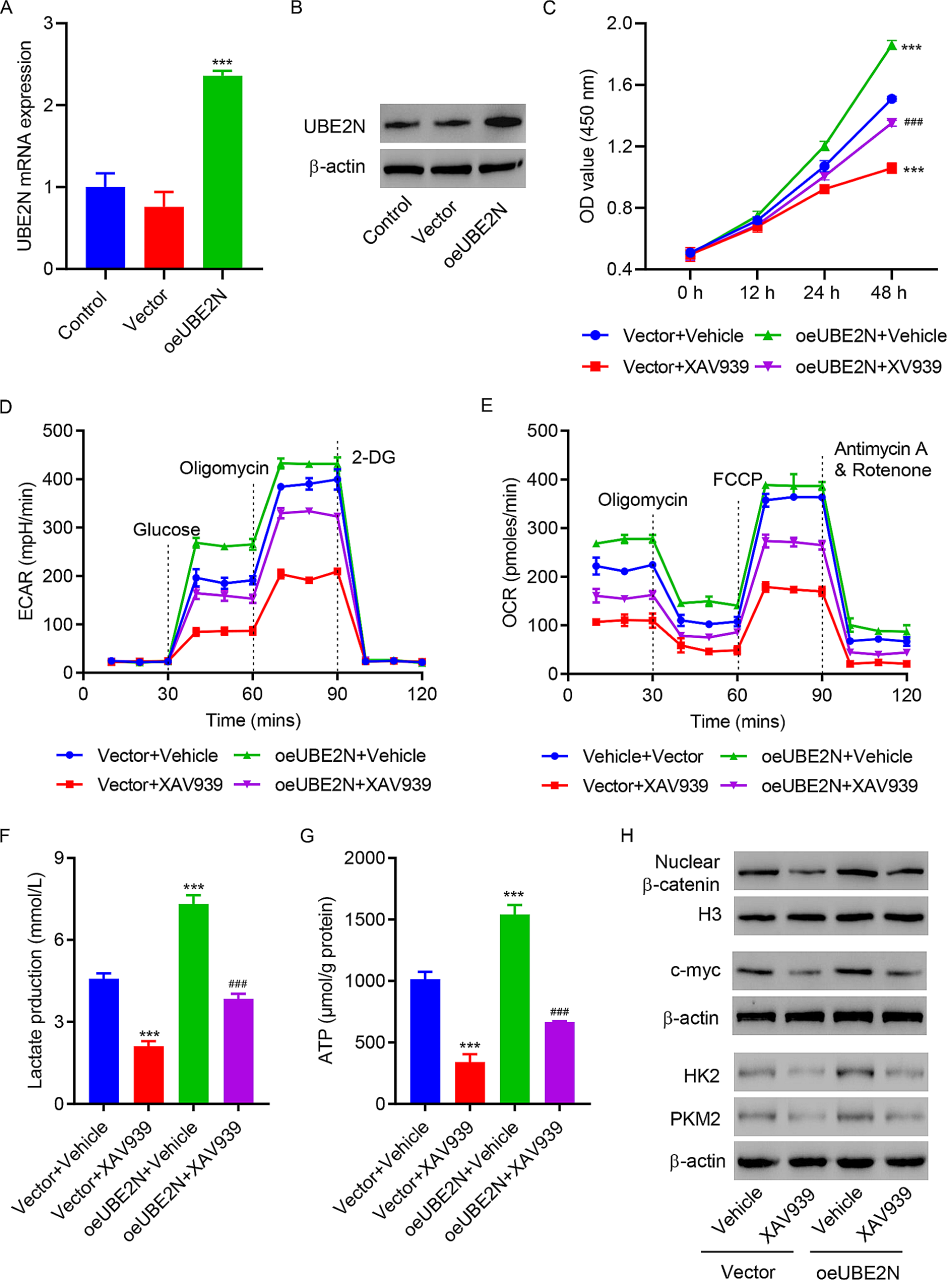
UBE2N overexpression promotes viability and glycolysis in PC3 cells via the Wnt/β-catenin signaling pathway. **(A)** RT-qPCR and **(B)** Western blot assay showed UBE2N expression levels in PC3 cells transduced with either UBE2N expression or blank vector. PC3 cells were transduced with either UBE2N expression or blank vector and co-treated with XAV939 or vehicle. **(C)** Cell viability, **(D)** ECAR, **(E)** OCR, **(F) **lactate, **(G)** ATP production, and **(H)** expression of c-myc, nuclear β-catenin, HK2 and PKM2. Data are expressed as mean ± SD (*n* = 3). ***p < 0.001 vs. Vector + Vehicle; ^###^*p* < 0.001, vs. oeUBE2N + Vehicle

To explore the role of Wnt/β-catenin signaling in the functional mechanism of UBE2N, PC3 prostate cancer cells were overexpressed UBE2N (Fig. 4A and B) and treated with or without XAV939, an inhibitor for the Wnt/β-catenin pathway. UBE2N overexpression significantly augmented cell viability, ECAR, OCR, lactate, ATP production, and expression of c-myc, nuclear β-catenin, HK2 and PKM2 while these increases were reversed by the treatment of XAV939 (Fig. 4C and H), suggesting that the function of UBE2N is mediated by the Wnt/β-catenin pathway.

### UBE2N promotes Axin1 ubiquitination and degradation

**Fig. 5:**
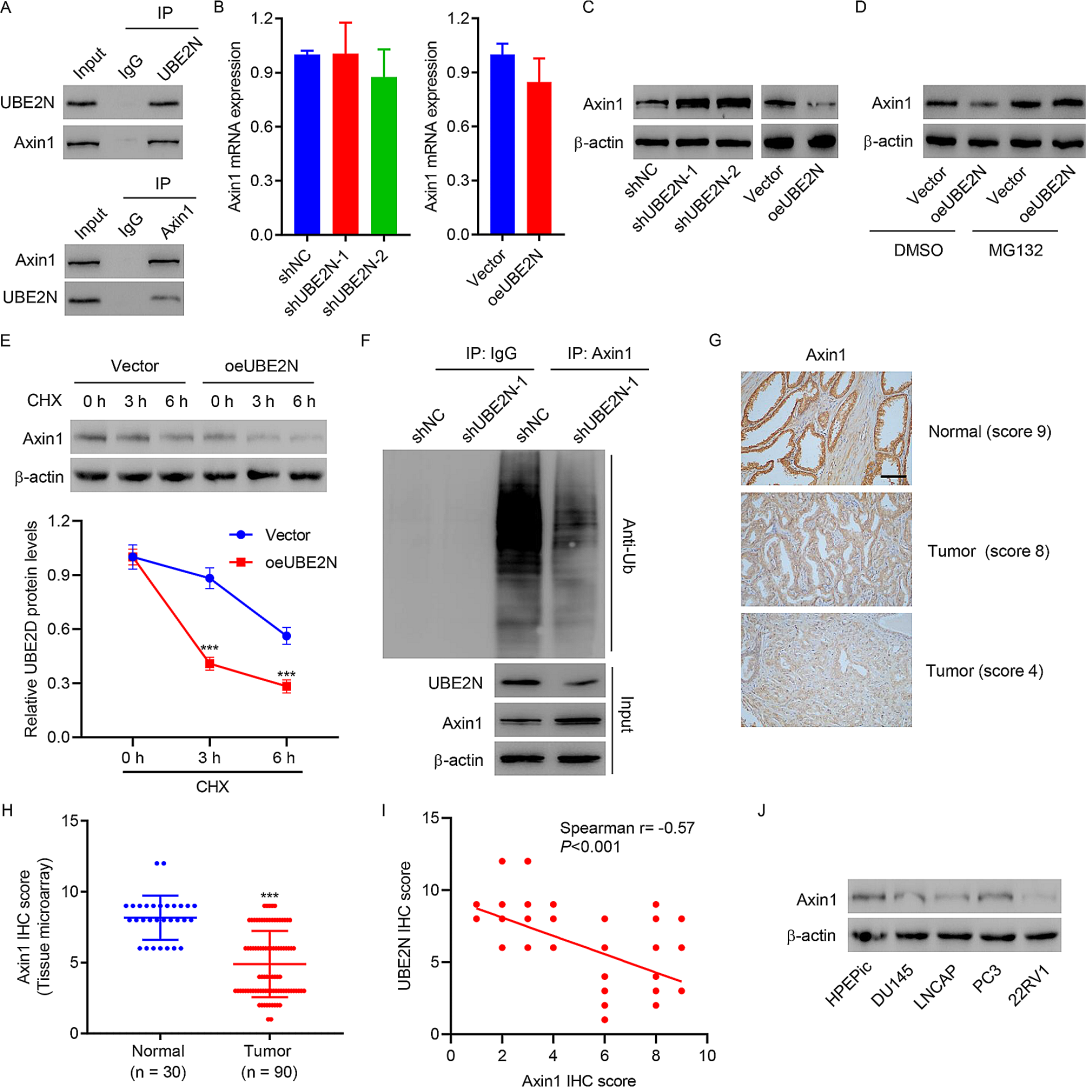
UBE2N interacts with Axin1 and results in its ubiquitination. **(A)** Cell lysates were subjected to immunoprecipitation with control IgG, anti-UBE2N, or anti-Axin1 antibody. The immunoprecipitates were then blotted with the indicated antibodies. **(B)** Axin1 mRNA expression was determined in 22RV1 cells transduced with shUBE2N-1 or -2 and in PC3 cells overexpressing UBE2N. Data are expressed as mean ± SD (*n* = 3). **(C)** Axin1 protein expression was determined in 22RV1 cells transduced with shUBE2N-1 or -2 and in PC3 cells overexpressing UBE2N. **(D, E)** Axin1 protein expression in PC3 cells overexpressing UBE2N in the absence/presence of MG132 or CHX. **(F)** 22RV1 cells transduced with shUBE2N-1 or shNC. Axin1 was immunoprecipitated and immunoblotted with the indicated antibodies. **(G, H)** Analysis of UBE2N protein in prostate cancer tissue microarray by IHC staining (scale bar, 100 μm). **(I)** Correlation analysis of UBE2N and Axin1 protein expression in prostate cancer tissue microarrays. **(J) **Axin1 protein levels were examined using Western blot. ***p < 0.001 vs. normal or vector

To investigate how UBE2N activates β-catenin pathway, the interaction between UBE2N and the β-catenin destruction complex including Axin1, GSK-3β and APC was examined by Co-IP. As shown in Fig. 5A and S2, UBE2N co-immunoprecipitated with Axin1 but not GSK-3β and APC. Reciprocal immunoprecipitation with Axin1 antibody also brought down UBE2N (Fig. 5A), suggesting an interaction between UBE2N and Axin1 in cells. In 22RV1 cells with UBE2N knockdown, the Axin1 protein level was increased but the Axin1 mRNA expression level was not changed significantly (Fig. 5B and C). In PC3 cells with UBE2N overexpression, the Axin1 protein level was decreased but Axin1 mRNA expression was not changed (Fig. 5B and C). In addition, the decrease in Axin1 level induced by UBE2N overexpression could be reversed by the addition of proteasome inhibitor MG132, suggesting that UBE2N regulates Axin1 levels in a proteasome-dependent manner (Fig. 5D). To further establish that UBE2N regulates Axin1 stability, we treated PC3 cells with cycloheximide (CHX) and determined the half-life of Axin1. As shown in Fig. 5E, Axin1 stability was dramatically decreased in UBE2N overexpressed cells, suggesting that UBE2N destabilizes Axin1. We next examined whether UBE2N regulates the level of Axin1 ubiquitination. As shown in Fig. 5F, UBE2N silencing resulted in a significant decrease in polyubiquitination of Axin. These results suggest that UBE2N ubiquitinates Axin1 in prostate cancer cells. Furthermore, Axin1 protein expression in prostate cancer tissue microarrays was also examined by IHC staining (Fig. 5G). Axin1 protein expression was decreased remarkably in tumor specimens when compared to normal tissues (Fig. 5H). Correlation analysis showed that UBE2N protein expression was negatively correlated with Axin1 protein expression in patients with prostate cancer (Fig. 5I). Moreover, the Axin1 protein expression was also detected in DU145, LNCAP, PC3, and 22RV1 prostate cancer cell lines (Fig. 5J).

#### Overexpressed Axin1 abrogates the promotive action of UBE2N overexpression on cell viability and glycolysis

**Fig. 6:**
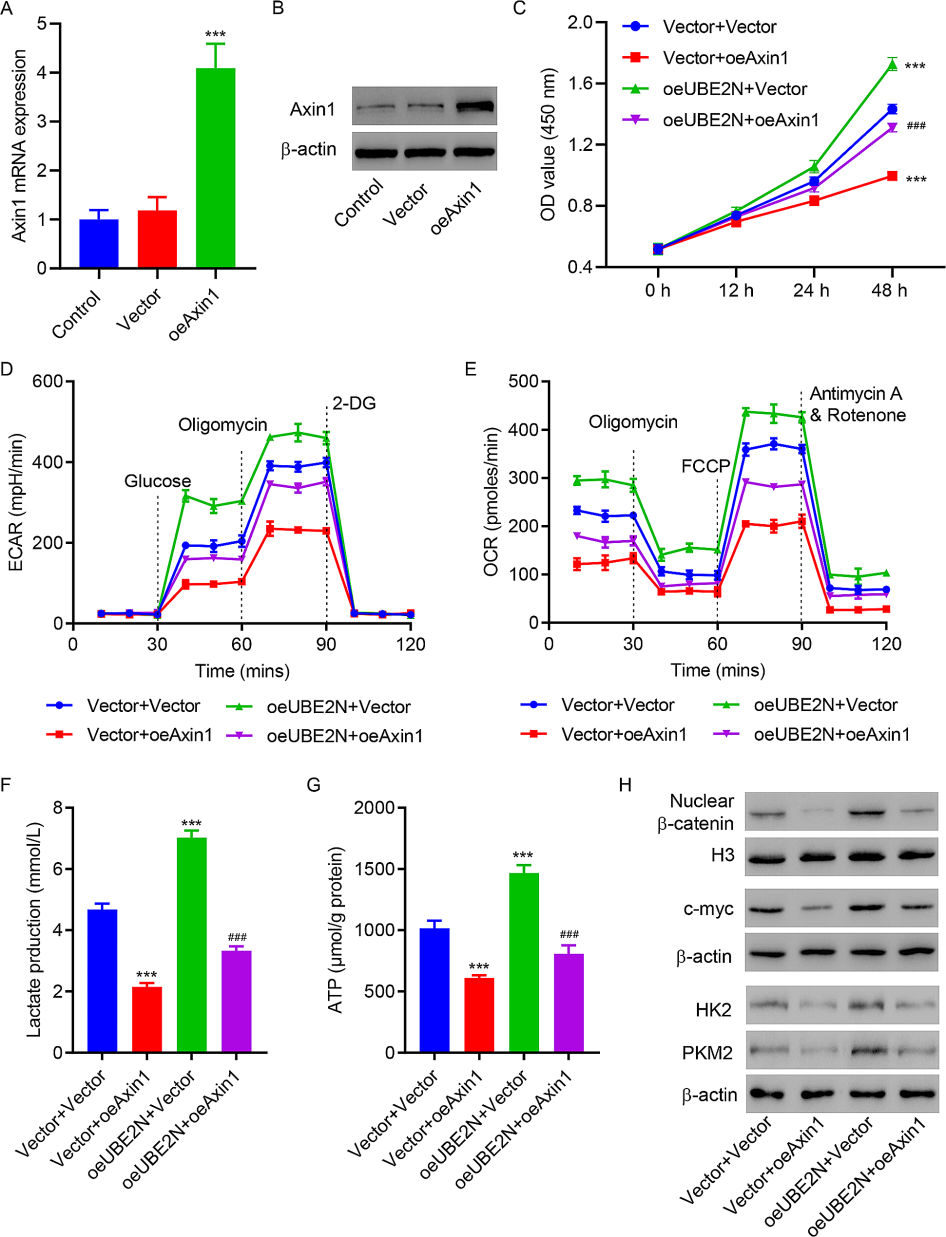
Axin1 overexpression abrogates the effects of UBE2N overexpression on PC3 cells. **(A)** RT-qPCR and **(B)** Western blot assay showed Axin1 expression levels in PC3 cells transfected with Axin1 expression vector or blank vector. PC3 cells were transfected with Axin1 expression vector and transduced with UBE2N expression vector. **(C)** Cell viability, **(D)** ECAR, **(E)** OCR, **(F)** lactate, **(G)** ATP production, and **(H)** expression of c-myc, nuclear β-catenin, HK2 and PKM2. Data are expressed as mean ± SD (*n* = 3). ***p < 0.001 vs. Vector + Vector; ^###^*p* < 0.001, vs. oeUBE2N + Vector

To examine the role of Axin1 in UBE2N-induced cell viability and glycolysis, PC3 cells were transfected with Axin1 expression vector (Fig. 6A and B). Figure 6C showed that co-expression of Axin1 and UBE2N inhibited cell viability as compared with UBE2N overexpression alone. Figure 6D and G showed that co-expression of Axin1 and UBE2N suppressed ECAR, OCR, lactate and ATP production as compared with UBE2N overexpression alone. Western blotting showed that UBE2N-induced increase in expressions of c-myc, nuclear β-catenin, HK2 and PKM2 were abrogated by Axin1 overexpression (Fig. 6H).

## Discussion

**Fig. 7:**
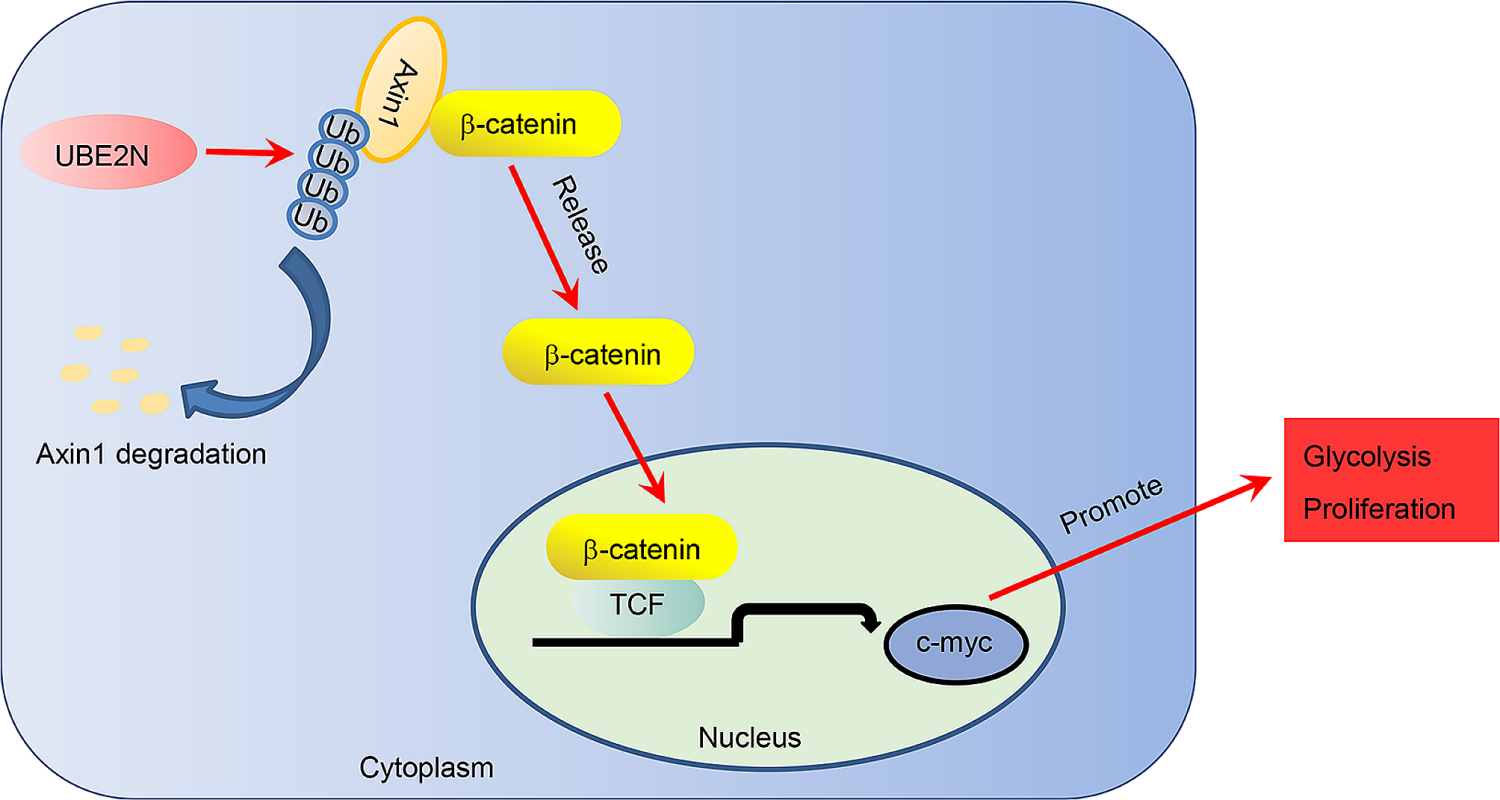
A diagram of the interaction of UBE2N and Axin1/Wnt/β-catenin signaling in regulating prostate cancer cell viability and glycolysis

With an increasing understanding of the biological process in cancer cell survival, some studies support the critical role of ubiquitin ligases that destabilize intracellular protein levels in highly proliferating cancer cells. Our current work highlighted abnormally high expression of ubiquitin ligase UBE2N in prostate cancer cells associated with poor prognosis and cell viability and glycolysis. Mechanically, UBE2N increases Axin1 ubiquitination and reduces Axin1 protein level, which subsequently facilitates β-catenin entry into the nucleus and activates Wnt/β-catenin signaling (Fig. 7).

Proteomics and IHC analysis on cancer tissue specimens revealed abnormal UBE2N protein expression [21, 22]. Davalieva K et al. found dysregulation of UBE2N in prostate cancer tissue specimens by gel electrophoresis coupled with mass spectrometry [23]. Singh AN et al. identified UBE2N as a contributor to cancer development by quantitative proteomic mass spectrometry profiling and gene enrichment analysis on the induced invasive phenotype of prostate cancer cells [24]. These two studies foremost examined the association between UBE2N and prostate cancer, but the elucidation of UBE2N function on prostate cancer cells remains elusive. Given the reported action of UBE2N on cancer progression in the literature [25, 26], we explored the effect of this gene on prostate cancer cell growth. In this study, we observed that UBE2N exerted an active effect on cell viability and tumor growth.

We have a remarkable finding that UBE2N promotes glycolysis in prostate cancer cells, evidenced by increased ECAR, ATP and lactate production. One of the marked characteristics of cancer cells is that large amounts of glycolysis and lactic acid production occur under aerobic conditions, which can provide energy and intermediate production of nucleotides or amino acids for the high viability of cells [27]. Our data also demonstrated that UBE2N knockdown decreased cell viability and OCR with a concomitant decrease in basal, maximal and ATP-linked respiration. Basal respiration is usually tightly regulated by ATP turnover and also partially by substrate oxidation and proton leak [28]. Increased basal respiration indicates elevated ATP demand, which is potentially linked to higher cell viability [29]. Therefore, UBE2N may mediate basal respiration rate by regulating ATP production and cell viability. Previous studies have considered UBE2N as a promising treatment target for intervention in cell viability in some kinds of cancers [26, 30]. However, little is understood about the mechanisms by which UBE2N acts on cancer cell viability. Our work suggested that in prostate cancer cells with UBE2N knockdown, glycolysis rate and ATP level were also significantly decreased. Therefore, we hold the opinion that UBE2N acts as a promoter for prostate cancer cell viability via enhancing intracellular glycolysis.

It is generally recognized that UBE2N seems to be critical for immune and inflammation responses due to its catalytic K63-linked polyubiquitination modification function [31, 32]. In previous studies associated with cancer progression, UBE2N mostly functioned on the activation/inactivation of cancer-related signaling pathways (e.g., MEK/FRA1/SOX10 and TAK1-p38 MAP kinase cascade) [25, 33]. However, little information has been recorded about the targets of UBE2N ubiquitination modification in cancer cells. In our study, UBE2N binds and destabilizes Axin1 but does not affect its transcription, suggesting ubiquitination is the major regulation of Axin1 by UBE2N.

Our results comprehensively elucidate the interaction among the Wnt/β-catenin pathway, Axin1, and UBE2N in cell viability and glycolysis. Extensive works have shown that the degradation of Axin1 is closely correlated with Wnt/β-catenin pathway activation [18]. Axin1 ubiquitination, degradation and subsequent liberation of β-catenin nuclear translocation are closely correlated with the activation of the expression of numerous genes that participate in most of the mechanism that leads to the development and progression of cancers [34]. We are concerned that the Wnt/β-catenin pathway is considered a contributor to cell viability and glycolysis in several cancers [35–37]. In our data, the expression of HK2, PKM2, c-myc and β-catenin, ATP production, and lactate production induced by UBE2N overexpression was abrogated by Axin1 overexpression. As reported in previous studies, activation of Wnt/β-catenin signaling or its target gene c-myc in prostate cancer promoted tumor progression, while inactivating Wnt/β-catenin signaling restricted tumor growth [10, 11]. Glycolytic enzymes, such as HK2 and PKM2, were upregulated to stimulate glycolysis in a c-myc-dependent manner [38]. These data suggested that UBE2N/Axin1/Wnt/β-catenin forms a signaling axis in controlling the viability and glycolysis of prostate cancer cells.

In conclusion, the present results emphasize UBE2N functions in regulating glycolysis and viability of prostate cancer cells. Further mechanism study points out that Axin1 is a target of the UBE2N ubiquitination modification function and is responsible for activating the Wnt/β-catenin pathway. Our findings approve UBE2N as a promising treatment target for prostate cancer.

### Electronic supplementary material

Below is the link to the electronic supplementary material.

Supplementary Material 1

## Data Availability

No datasets were generated or analysed during the current study.
