# Supplementary material for: UBE2N promotes cell viability and glycolysis by promoting Axin1 ubiquitination in prostate cancer cells
Source: Biol Direct. 2024 May 7;19:35. doi: 10.1186/s13062-024-00469-y (PMC11075218; doi:10.1186/s13062-024-00469-y)
Supplement: Supplementary file 1 — Supplementary Material 1 [file 13062_2024_469_MOESM1_ESM.docx]

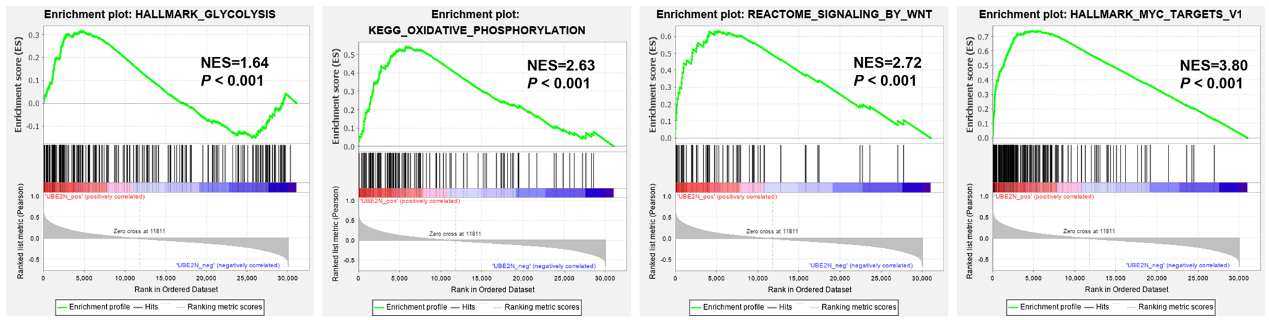


**Figure S1**. GSEA of UBE2N associated with “HALLMARK_GLYCOLYSIS”, “KEGG_OXIDATIVE_PHOSPHORYLATION”, “REACTOME_SIGNALING_BY_WNT”, or “HALLMARK_MYC_TARGETS_V1” in patients with prostate cancer.


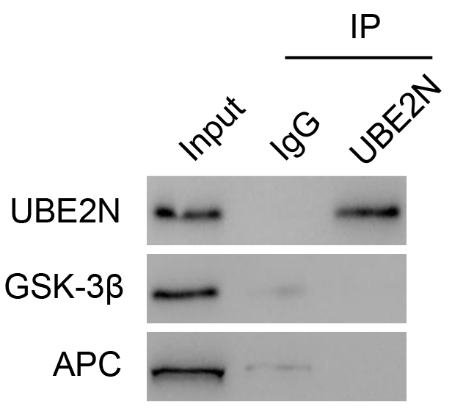


**Figure S2**. Co-IP assay showed no binding between UBE2N and GSK-3β or APC.
